# Clinical neurophysiology of REM parasomnias: Diagnostic aspects and insights into pathophysiology

**DOI:** 10.1016/j.cnp.2023.10.003

**Published:** 2024-01-10

**Authors:** Melanie Bergmann, Birgit Högl, Ambra Stefani

**Affiliations:** a Department of Neurology, Sleep Laboratory, Medical University Innsbruck, Austria; b Neurological Clinical Research Institute, Massachusetts General Hospital, Boston, USA

**Keywords:** RBD, REM sleep, REM sleep behavior disorder, Recurrent isolated sleep paralysis, Nightmare disorder, Electroencephalography, Electrooculography, Electromyography

## Abstract

Parasomnias are due to a transient unstable state dissociation during entry into sleep, within sleep, or during arousal from sleep, and manifest with abnormal sleep related behaviors, perceptions, emotions, dreams, and autonomic nervous system activity.

Rapid eye movement (REM) parasomnias include REM sleep behavior disorder (RBD), isolated recurrent sleep paralysis and nightmare disorder. Neurophysiology is key for diagnosing these disorders and provides insights into their pathophysiology.

RBD is very well characterized from a neurophysiological point of view, also thank to the fact that polysomnography is needed for the diagnosis. Diagnostic criteria are provided by the American Academy of Sleep Medicine and video-polysomnography guidelines for the diagnosis by the International REM Sleep Behavior Disorder Study Group. Differences between the two sets of criteria are presented and discussed. Availability of polysomnography in RBD provides data on sleep electroencephalography (EEG), electrooculography (EOG) and electromyography (EMG). Sleep EEG in RBD shows e.g. changes in delta and theta power, in sleep spindles and K complexes. EMG during REM sleep is essential for RBD diagnosis and is an important neurodegeneration biomarker. RBD patients present alterations also in wake EEG, autonomic function, evoked potentials, and transcranial magnetic stimulation.

Clinical neurophysiological data on recurrent isolated sleep paralysis and nightmare disorder are scant. The few available data provide insights into the pathophysiology of these disorders, demonstrating a state dissociation in recurrent isolated sleep paralysis and suggesting alterations in sleep macro- and microstructure as well as autonomic changes in nightmare disorder.

## Highlights

• RBD presents REM sleep without atonia, EEG slowing, impaired functional connectivity, microstructural sleep EEG changes, among others.
• During sleep paralysis there is a REM-wake dissociation - an intermediate state between REM and wakefulness, REM sleep or EEG alpha frequencies.
• Nightmare disorder shows reduced sleep, increased awakenings, reduced SWS, increased respiratory and heart rate, and high alpha power in REM sleep.

## 1 Introduction

The word parasomnia derives from the combination of the ancient Greek word παρά (pará), which means “at,” “in,” “during,” “next to” and the Latin word somnus, “the sleep”. Parasomnias are adverse physical events or experiences that occur during entry into sleep, within sleep, or during arousal from sleep, manifesting with abnormal sleep related complex movements, behaviors, perceptions, emotions, dreams, and autonomic nervous system activity.

There are three states of human consciousness, i.e. wake, non rapid eye movement (NREM) sleep and rapid eye movement (REM) sleep. A complex orchestration of several central nervous system areas and pathways ensures a stable state declaration under physiological conditions. However, during sleep-wake cycles oscillations, the state may not be fully declared leading to a transient unstable state dissociation with combinations of one or more of these states. Parasomnias are the results of such state dissociation.

Parasomnias are classified into NREM sleep and REM-sleep parasomnias. This review will focus on REM sleep parasomnias, which according to the International Classification of Sleep Disorders, third edition text revision (ICSD-3 TR), include REM sleep behavior disorder (RBD), recurrent isolated sleep paralysis and nightmare disorder.

Neurophysiology plays a relevant role in these diseases, as neurophysiological techniques are essential for diagnostic purposes in RBD, and helpful in differentiating all three REM-sleep parasomnias from other disorders, as well as in providing insight into the pathophysiology of these sleep disorders.

Neurophysiological features of RBD are well characterized due to the availability of video-polysomnographic recordings (required for diagnosing the disease), which include electroencephalography (EEG), electrooculography (EOG) and electromyography (EMG) signals. In addition, the high interest in isolated RBD (iRBD) as prodromal synucleinopathy lead to numerous studies assessing neurophysiological data during daytime, such as EEG, autonomic function, evoked potentials and transcranial magnetic stimulation.

Only a small number of neurophysiological studies on recurrent isolated sleep paralysis and nightmare disorder are available. These data will be presented and discussed in the following sections.

## 2 REM sleep behavior disorder

### 2.1 Definition of REM sleep behavior disorder (RBD)

RBD is a REM sleep parasomnia characterized by dream enactment behaviors and REM sleep without atonia (RWA). Secondary RBD (i.e., RBD associated to other conditions like overt neurodegenerative disease, narcolepsy, autoimmune diseases, stroke) can emerge in adults before age 50 years, and affects both sexes without relevant differences. iRBD (i.e., not associated to other conditions) is more frequent in males and after 50 years of age, and represents an early phase synucleinopathy, phenoconverting over time into dementia with Lewy Bodies, Parkinsons’s disease or multiple system atrophy (Stefani et al., 2023). In a *meta*-analysis, the rate of conversion into neurodegenerative disease was 33.5 % at five years follow-up, 82.4 % at 10.5 years and 96.6 % at 14 years (Galbiati et al., 2019).

Neurophysiology is key for diagnosing RBD. Diagnostic criteria for RBD are provided by the American Academy of Sleep Medicine (2023) (AASM) in the ICSD-3 TR, and video-polysomnography (VPSG) guidelines for RBD diagnosis are provided by the International REM Sleep Behavior Disorder Study Group (IRBDSG). According to the ICSD-3 TR, diagnosis of RBD requires I. repeated episodes of sleep-related vocalizations and/or complex motor behaviors, which are documented by VPSG to occur during REM sleep, or are presumed to occur during REM sleep based on clinical history of dream enactment, and II. the presence of RWA. The IRBDSG VPSG guidelines instead require VPSG to demonstrate not only the presence of RWA, but also to document at least one RBD episode (Cesari et al., 2022). In addition, the IRBDSG VPSG guidelines specify technical requirements for RBD diagnosis and recommendations on how to score REM sleep in the context of RBD in case it is not possible to apply the AASM criteria (Cesari et al., 2022), e.g. in patients with neurodegenerative diseases.

According to the AASM scoring manual version 3, RWA requires during REM sleep: excessive sustained muscle activity (i.e., tonic activity) in the chin EMG, excessive transient muscle activity (i.e., phasic activity) in the chin or limb EMG, or at least 50 % of 3 s mini-epochs containing any chin or limb EMG activity (Troester et al., 2023). The IRBDSG recommended REM sleep scoring in 3-s mini epochs, followed by quantification of chin (tonic, phasic and any) and flexor digitorum superficialis (FDS) phasic EMG activity, to obtain a combined index of any chin and/or phasic FDS EMG activity according to the SINBAR (Sleep INnsbruck BARcelona) scoring method (Frauscher et al., 2012a). Moreover, the IRBDSG VPSG guidelines provide some additional recommendations for RWA quantification, in order to achieve reliable scoring: at least five minutes of REM sleep are required, and the apnea-hypopnea-index during REM sleep needs to be below 15/h (i.e., in case of sleep apnea this needs to be treated prior to RWA quantification) (Cesari et al., 2022). A comparison between the current AASM scoring manual and the IRBDSG VPSG guidelines for quantification of RWA is provided in Table 1.

**Table 1:** Comparison between the American Academy of Sleep Medicine (AASM) scoring manual Version 3 (Troester et al., 2023) and the International REM Sleep Behavior Disorder Study Group (IRBDGS) (Cesari et al., 2022) video-polysomnography guidelines for quantification of rapid eye movement (REM) sleep without atonia (RWA).

	AASM Version 3 2023	IRBDSG 2022
REM sleep	Epochs containing RWA with tonic chin activity can be scored as R if other criteria for stage R are met or if the epoch is contiguous with an epoch scored as stage R	Score R in presence of characteristic finding on at least 2/4 parameters (EEG, EOG, EMG and synchronized audiovisual recordings)
Time-synchronized video + audio	Recommended for RBD diagnosis	Mandatory for RBD diagnosis Technical recommendations provided Scoring recommendations provided
EMG technical requirements		
Sampling rates	500 Hz	1000 Hz
Filtering	10–100 Hz	5–500 Hz
Minimum digital resolution	12 bits per sample	16 bits per sample
EMG montage	For RWA detection, use FDS or EDC (optional)	EMG recording of the upper extremities (FDS) recommended

Abbreviations: AASM: American Academy of Sleep Medicine scoring manual Version 3; IRBDGS: International REM Sleep Behavior Disorder Study Group; REM: Rapid eye movement; R: REM; RWA: REM sleep without atonia; EEG: electroencephalography; EOG: electrooculography; EMG: electromyography; RBD: REM sleep behavior disorder; FDS: Flexor digitorum superficialis; EDC: extensor digitorum communis.

### 2.2 Neurophysiology of RBD

#### 2.2.1 Video-polysomnography

##### 2.2.1.1 Sleep electroencephalography

EEG slowing is present in patients with iRBD not only during wakefulness (as discussed later, in 2.2.2) but also during sleep, with higher amount of delta and theta power in EEG spectral analysis compared to controls (Fantini et al., 2003, Massicotte-Marquez et al., 2008). EEG power spectrum analysis during REM sleep have been reported to show a lower degree of difference from stage N2 a higher degree of instability in iRBD compared to controls (Ferri et al., 2017).

Several studies looked into differences in EEG during REM sleep between RBD patients and controls. One study investigating EEG spectral analysis found marked EEG slowing with increased delta and theta activity in the central and occipital regions during wakefulness and REM sleep (particularly in the right hemisphere) in patients with RBD and mild cognitive impairment (MCI) compared to controls, and to a lesser extent in patients with RBD and MCI compared to iRBD without MCI. Of note, the described EEG pattern was comparable to those of patients with dementia with Lewy bodies and Parkinson’s disease dementia (Iranzo et al., 2010), suggesting that these EEG findings may relate to later development of MCI or dementia in patients with iRBD. In line with these findings, subsequent studies underlined the possible association between EEG slowing during REM sleep and wakefulness on the one side, and neurodegeneration leading to cognitive impairment in RBD as well as dementia and Parkinson’s disease on the other side (Sasai et al., 2013, Rodrigues Brazete et al., 2016). Besides EEG slowing, during REM sleep an increased arousal index has been reported in iRBD patients and patients with Parkinson’s disease and RBD compared to healthy controls, as well as changes in arousals’ characteristics with a lower shift in α-band power at arousals and a higher muscle tone during arousals in iRBD and Parkinson’s disease with RBD compared to healthy controls (Brink-Kjaer et al., 2021).

Furthermore, a lack of suppression of beta rhythms during phasic REM sleep (i.e, REM sleep with bursts of rapid eye movements) has been reported in RBD patients. (Valomon et al., 2021). The authors suggest that a blunted difference between REM sleep sub-stages may constitute a sensitive biomarker for RBD.

EEG spectral power analysis showed that, after a mean follow-up duration of 6.64 years, iRBD phenoconverters during phasic REM sleep had significantly higher delta and lower alpha power, mainly in the central and occipital regions, compared to nonconverted iRBD (Gong et al., 2022).

Reported EEG findings during REM sleep in iRBD are likely related to the ongoing alpha-synuclein related neurodegeneration, in particular with EEG slowing correlating to cognitive function and arousal changes suggesting neurodegeneration affecting the reticula activation system.

For what concerns EEG changes during NREM sleep in RBD, data are less clear. Some studies found no spectral power changes (Latreille et al., 2011, Sasai et al., 2013) and no difference in slow wave activity (Latreille et al., 2011) between patients with iRBD and controls, whereas other reported an increased delta power (Massicotte-Marquez et al., 2005) or a diffuse reduction in alpha power in patients with RBD compared to healthy controls (Sunwoo et al., 2021).

Further changes reported in sleep EEG in patients with iRBD include alterations in sleep spindles and slow oscillations (SO). SO have been found to have lower amplitude, longer duration and shallower morphology and the spindles power (based on the peak of the spindle envelope) was lower in patients with iRBD compared to controls (Sunwoo et al., 2021). Another study found significantly lower densities of sleep spindles in patients with RBD compared to controls. However, further analysis showed lower densities of fast spindles and higher densities of slow spindles (O'Reilly et al., 2015). Similarly, a reduction in spindle density was found in both iRBD and Parkinson’s disease with RBD compared to healthy controls (Christensen et al., 2014). Moreover, a reduction in K-complex density was described in iRBD patients with MCI compared to those without MCI (Galbiati et al., 2021). Changes in sleep spindles and K-complexes are thus potential markers of neurodegeneration.

High density (256-channel) EEG in nine patients with iRBD and two matched control groups showed no significant decline in slow wave activity from early to late NREM sleep, mostly driven by lower slow wave activity in RBD patients at the beginning of the night, in particularly in the frontal regions. Moreover, in RBD patients the decline in slow wave amplitude from early to late sleep was less pronounced than in controls (Valomon et al., 2021). Overall, these changes suggest an impaired NREM sleep homeostasis in RBD.

The cyclic alternating pattern (CAP) is a periodic EEG activity during NREM sleep, characterized by sequences of transient recurrent electrocortical events, expressing sleep instability. CAP changes in patients with RBD compared to controls had been reported, suggesting an involvement of NREM microstructure in RBD (Kutlu et al., 2013, Melpignano et al., 2019). However, the two studies investigating CAP in iRBD reported contrasting findings, i.e. higher CAP cycles, CAP sequences, CAP indices and CAP rates in one (Kutlu et al., 2013), decreased CAP rate and CAP in the other one (Melpignano et al., 2019).

Taken together, sleep EEG findings in RBD suggest that some EEG features are likely related to more advanced neurodegeneration and may be considered biomarkers of phenoconversion in iRBD.

##### 2.2.1.2 Electrooculogram

Rapid eye movements (REMs) were first described by Aserinsky and Kleitman (Aserinsky and Kleitman, 1953, Aserinsky and Kleitman, 1955) and are mainly associated with motor activity during REM sleep in RBD (Frauscher et al., 2009, Leclair-Visonneau et al., 2010, Manni et al., 2009). Of note, in 20 subjects with RBD REMs, isolated or in bursts, were temporally associated with both emotional and neutral behaviors (Maranci et al., 2022).

The oculomotor system may be affected in patients with neurodegenerative disorders, and could be affected in prodromal stages such as RBD as eye movement are partially controlled by brainstem neurons. In line with this, a significantly lower frequency of nocturnal eye movements during nocturnal wakefulness and significantly higher frequency of eye movements during N2 sleep was reported in patients with iRBD as well in patients with Parkinson’s disease and RBD (Christensen et al., 2021).

##### 2.2.1.3 Electromyography

The first description of tonic EMG activity during REM sleep in patients with Parkinson’s disease was published in 1969 (Traczynska-Kubin et al., 1969). In 1975, Mouret reported in 13 out of 23 patients with Parkinson’s Disease a persistence of EMG activity of the chin during paradoxical sleep, occurrence of rapid eye movements, blepharospasms during slow wave sleep before the onset of paradoxical sleep episodes, and alpha rhythm during paradoxical sleep (Mouret, 1975). Tachibana and colleagues introduced the term “stage-1 REM with tonic EMG activity” in 1975 (Tachibana et al., 1975) and Guilleminault et al. described RWA as “sleep stage 7” (Guilleminault et al., 1976). The term REM sleep behavior disorder in humans was first proposed by Schenk et al. in 1986, when reporting a case series of five patients with variable loss of chin atonia, increased limb-twitch activity, increased REM ocular activity and density as well as REM sleep behaviors recorded on videotape (Schenck et al., 1986).

Lapierre and Montplaisir firstly developed a scoring method to quantify RWA in 1992 (Lapierre and Montplaisir, 1992). From this starting point, several centers developed their own scoring method. An overview is provided in Cesari et al., Table 1 (Cesari et al., 2022). Initially, RWA was quantified in the mentalis and submentalis muscles due to their routine use during polysomnography, but several studies reported a reduced sensitivity in diagnosing RBD using chin EMG only (Fernandez-Arcos et al., 2017). Iranzo and colleagues reported that a simultaneous recording of mentalis, FDS and extensor digitorum brevis muscles EMG in eleven patients with iRBD detected 94.4 % of motor and vocal manifestations during RBD episodes (Iranzo et al., 2011). Subsequent works by the SINBAR group provided further support to the recommendation of using a VPSG montage including the upper extremities to improve diagnostic accuracy of RBD (Frauscher et al., 2012a, Frauscher et al., 2014). In particular, discriminative power for the diagnosis of RBD was higher in the upper extremities compared to lower limb muscles (Frauscher et al., 2012a), and in nine patients with REM sleep atonia the quantification of EMG activity in the mentalis muscle and upper limbs, increased the ability to diagnose idiopathic RBD (Fernandez-Arcos et al., 2017).

**Fig. 1:**
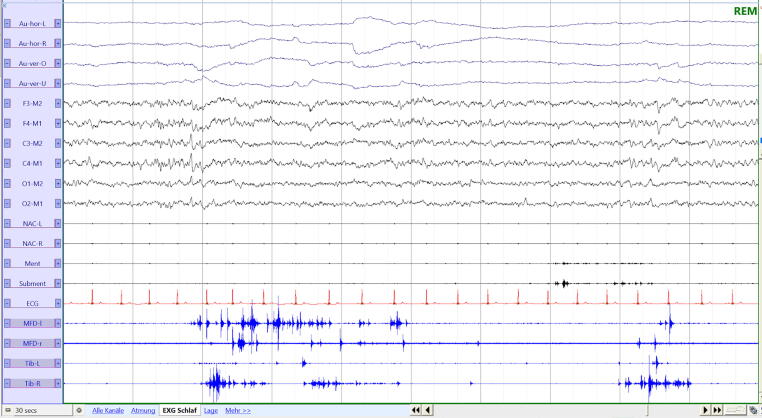
REM sleep without atonia. The figure shows a 30-s polysomnography page of a RBD patients with REM sleep without atonia. The electrooculography channels (Au) show rapid eye movements. The EEG channels (F3-M2, F4-M1, C3-M2, C4-M1, O1-M2, O2-M1) show the low-amplitude mixed frequency activity typical of REM sleep. The EMG channels show excessive muscle activity in particular in the limbs. Abbreviations: Au-hor-L: Electrooculography horizontal left; Au-hor-R: electrooculography horizontal right; Au-ver-L: Electrooculography vertical left; Au-ver-R: electrooculography vertical right; ECG: electrocardiography; EEG, electroencephalography; ment: muscle mentalis; MFD-L: muscle flexor digitorum superficialis left; MFD-R: muscle flexor digitorum superficialis right; NAC-L: muscle stenocleidomastoideus left; NAC-R: muscle stenocleidomastoideus right; subment: muscle submentalis; Tib-L: muscle tibialis anterior left; Tib-R: muscle tibialis anterior right.

The current version of the AASM Manual for the Scoring of Sleep and Associated Events (Troester et al., 2023) and the IRBDSG VPSG guidelines (Cesari et al., 2022) both refer to the SINBAR criteria for quantifying RWA (Table 1). An example of a 30-second VPSG page showing RWA is presented in Fig. 1.

Besides RWA, which is the neurophysiologic hallmark of RBD, in these patients the EMG signal can reveal excessive fragmentary myoclonus (EFM) as phasic EMG activity of the tibialis anterior muscles, which is more frequent in REM sleep in general and was reported to be present in a higher proportion of iRBD compared to controls (Nepozitek et al., 2019, Stefani and Hogl, 2019).

#### 2.2.2 Further neurophysiological aspects in RBD

Assessing movements, actigraphy is a reliable measure of sleep/wake cycles, but does not allow sleep staging. Actigraphy has been reported as an alternative and possibly faster method for identifying RBD, an is a reliable screening instrument (Stefani et al., 2018, Brink-Kjaer et al., 2023, Raschella et al., 2023). The number studies on actigraphy in RBD is still limited, but current data support use of actigraphy not only for screening purposes in RBD but also as marker of phenoconversion (Liguori et al., 2023) and to assess treatment response (Park et al., 2023).

#### 2.2.3 EEG findings in wakefulness

The most consistent EEG finding during wakefulness is a slowing in patients with iRBD compared to controls (Fantini et al., 2003, Sasai et al., 2013). Another study confirmed EEG slowing during wake in patients with iRBD and MCI, who had higher relative theta power in the parietal, temporal and occipital regions and lower relative alpha power in the occipital region compared to iRBD without MCI and controls. The authors found the dominant occipital frequency to be lower in patients with iRBD and MCI compared to controls and concluded that the EEG slowing in the posterior cortical regions could provide a marker for an increased risk of developing dementia with Lewy bodies or Parkinson’s disease (Rodrigues Brazete et al., 2013).

Additionally, functional resting state connectivity during relaxed wakefulness, measured as weighted phase lag index, showed a decreased delta-band functional connectivity in the frontal regions in iRBD compared to controls, possibly related to early neurodegeneration (Sunwoo et al., 2017).

Similarly, a high-density EEG study during relaxed wakefulness in 18 iRBD patients and healthy controls found an increased alpha phase synchronization in EEG bands and reduced delta band correlations (Roascio et al., 2022). The authors interpreted those results as an active mechanism to compensate cognitive impairment in people with iRBD.

Wake EEG changes during sleep can thus be considered a neurodegeneration marker, possibly a marker of disease, of progression, and of risk of phenoconversion.

#### 2.2.4 Autonomic function in RBD

Autonomic dysfunction is frequent in iRBD (Frauscher et al., 2012b, McCarter et al., 2020) and arises mainly from peripheral (e.g. lower resting heart rate and blood pressure variability) rather than from central autonomic dysregulation (e.g. baroreflex sensitivity, central cardiac autonomic outflow) (Dahms et al., 2016). Of note, more severe baseline cardiovagal autonomic dysfunction was associated with phenoconversion to dementia with Lewy bodies but not Parkinson’s disease (McCarter et al., 2020), and could therefore represent a marker of type of phenoconversion.

Heart rate variability, a measure of autonomic function, was impaired in RBD compared to controls and a reduced nocturnal heart rate variability correlated with autonomic impairment during wakefulness as well (Ferini-Strambi et al., 1996, Postuma et al., 2010, Sorensen et al., 2013).

Moreover, the heart rate response to leg movements was significantly lower in patients with Parkinson’s disease and iRBD compared to controls (Sorensen et al., 2012). Other autonomic functions that were reported to be impaired in RBD included sudomotor abnormalities (Zitser et al., 2020), impaired temperature sensation with higher thermal detection thresholds (Strobel et al., 2018), lower supine norepinephrine plasma levels and adversely affected cold and vibration detection threshold levels in quantitative sensory testing (Koch et al., 2022), and orthostatic hypotension and constipation (Eckhardt et al., 2023).

Autonomic dysfunction is common in iRBD, and specific findings may correlate with future phenoconversion to multiple system atrophy versus dementia with Lewy bodies versus Parkinson’s disease.

#### 2.2.5 Evoked potentials

Evoked potentials in RBD showed inconsistent results. One study investigating auditory evoked potentials (active and passive auditory oddball paradigms) found no differences between RBD and controls (Raggi et al., 2007). In contrast, another study reported that the P2 wave, an occipital positive wave, was present in patients with Parkinson’s disease and RBD but not in controls (Gaudreault et al., 2013). A few studies found higher rates of abnormalities in vestibular evoked myogenic potentials (VEMP) in iRBD and patients with RWA and early stage Parkinson’s disease compared to healthy controls (Xie et al., 2022, Puligheddu et al., 2019, de Natale et al., 2018), underlining the need for further studies in this area.

#### 2.2.6 Transcranial magnetic stimulation

Transcranial magnetic stimulation (TMS) is a neurophysiological technique of brain stimulation (Barker et al., 1985) used to identify deficits in brain inhibition and excitation (Rossini et al., 2015). Paired pulse TMS of several inhibitory and facilitatory circuits showed a non-significant reduction in short interval intracortical inhibition (SIGI) and a significant decrease in intracortical facilitation (ICF), mainly mediated by glutamatergic neurotransmission, in iRBD compared to controls, reflecting a significant hypofacilitation and a tendency towards a disinhibition of the motor cortex in iRBD (Lanza et al., 2020). A later study confirmed these findings, showing decreased ICF in both patients with iRBD and patients with RBD plus Parkinson’s disease (Lanza et al., 2022). It can be hypothesized that motor cortex disinhibition may facilitate behaviors during REM sleep.

Another measure of TMS, short latency inhibition (SAI), evaluating cholinergic circuits (Tokimura et al., 2000), was reduced in iRBD compared to controls and correlated with disease duration, episodic verbal memory and executive functions (Nardone et al., 2012). Thus, such changes may reflect more advanced neurodegeneration.

#### 2.2.7 Cortico-muscular coherence

Cortico-muscular coherence (CMC) describes the coherence ability between motor cortex and muscles via corticospinal pathways (Liu et al., 2019). Jung and co-workers investigated CMC in patients with RBD and found significantly higher CMC value in patients with RBD compared to controls, suggesting an increased cortical locomotor drive (Jung et al., 2012). Similarly, CMC and corticocortical coherence (CCC) were higher in patients with iRBD compared to controls (Choi et al., 2021).

Also here, it can be hypothesized that increased cortical locomotor drive may facilitate behaviors during REM sleep.

## 3 Recurrent isolated sleep paralysis

### 3.1 Definition of recurrent isolated sleep paralysis

Recurrent isolated sleep paralyses are characterized by the inability to perform voluntary movements and occur either at the onset of the sleep (hypnagogic) or upon awakening (hypnapompic). Respiratory movements are not affected and the environment is clearly perceived during the sleep paralysis. Sleep paralysis affects somatic muscles (Chase and Francisco, 1994) under voluntary control (Nielsen and Zadra) except the diaphragm, external eye muscles, and the stapedius muscle (Kryger et al., 1994) and can be accompanied by hypnagogic or hypnapompic (McCarty and Chesson, 2009) hallucinations (Högl and Iranzo, 2017). Sleep paralyses can be isolated but can also occur as a part of the clinical tetrad of narcolepsy, with a reported frequency of 50 % (Frauscher et al., 2013).

Mean age at onset of sleep paralysis is 14 to 17 years, without relevant sex differences. The average prevalence of at least one episode of sleep paralysis is 20.8 %. Irregular sleep-wake schedules and sleep deprivation have been identified as predisposing factors for the occurrence of sleep paralyses. The frequent occurrence of sleep paralysis, and their impact on people experiencing it, lead to several popular names describing this phenomenon: Sleep paralysis is also known as “the ghost oppression phenomenon” in Hong Kong (Wing et al., 1994) or was described in Mexico as “*Se me subio un muerto”* [“A cadaver climbed on me”] (Stefani et al., 2017). For the same reasons, sleep paralysis was also a topic in the fine arts. Johann Heinrich Füssli’s painting *The Nightmare* (1781) shows a sleep paralysis with hypnagogic hallucinations (Baumann et al., 2007). Additionally, sleep paralyses were also described in writings of great novelists like for instance in Dostoevsky’s “The Brothers Karamazov” (Stefani et al., 2017), in Gogol’s tale “The Portrait” (1833) (Aguirre et al., 2021) and in Maupassant’s “The Horla” (Miranda and Bustamante, 2015, Miranda and Högl, 2013).

### 3.2 Neurophysiology of recurrent isolated sleep paralysis

One early study reported the occurrence of sleep paralysis after acute reversal of the sleep-wake-cycle (Weitzman et al., 1970). Takeuchi and colleagues performed nocturnal sleep interruptions by forced awakening for 60 min in 16 healthy subjects. After sleep interruption, isolated sleep paralysis occurred in 9.4 % and sleep onset REM periods in 71.9 %. VPSG recordings during isolated sleep paralysis showed REM wake dissociated stage, with simultaneous alpha EEG and muscle atonia. The authors thus hypothesised that isolated sleep paralyses occur in the transition between REM sleep and wakefulness (Takeuchi et al., 1992).

Another polysomnography study included four people, three with suspected and one with known narcolepsy, and induced cataplectic events by emotional provocation. Unprovoked sleep paralysis occurred as well. Polysomnography during sleep paralysis showed REM sleep, but the study was based on four EEG channels, chin EMG and a single lower extremity channel only (Dyken et al., 1996). In contrast, a case report of a patient with narcolepsy documented an incomplete sleep paralysis via VPSG and showed that REM sleep ended before the beginning of the sleep paralysis. EEG during the episode revealed alpha activity with low-voltage fast activity. The chin EMG exhibited tonic and phasic muscle activity (Buskova et al., 2013). In another case report, sleep paralysis occurred on awakening from sleep onset REM period during the fourth multiple sleep latency test nap. The patient with narcolepsy type 1 (with cataplexy) was unable to move his limbs, had bilateral myosis and slowed/slurred speech. During the sleep paralysis the following features were detected: EEG with alpha frequencies with low-voltage fast activity, bursts of rapid eye movements, muscle atonia and loss of atonia of the submental EMG. After performing EEG frequency domain analysis (30-s EEG epochs) and fast Fourier transform frequency spectra calculations, the authors suggested that sleep paralysis could be an intermediate state between wake and REM sleep (Terzaghi et al., 2012). Other neurophysiological considerations are reported in a case-control study including 19 patients with recurrent isolated sleep paralysis. Power spectral analysis showed higher bifrontal beta activity during REM sleep. Macrostructural parameters of REM sleep revealed no differences between both groups (Klikova et al., 2021).

The neurophysiological characterization of sleep paralysis allows understanding of their pathophysiology, as sleep paralysis is an example of state dissociation with persistence of the muscle atonia typical of REM sleep in the waking state.

As possible differential diagnoses of sleep paralyses include focal epileptic seizures, assessment including both VPSG and prolonged video-EEG-monitoring should be performed in unclear cases (Galimberti et al., 2009, Bergmann et al., 2022).

## 4 Nightmare disorder

### 4.1 Definition of nightmare disorder

According to the ICSD-3 TR, the nightmare disorder is characterized by the repeated occurrence of extended, extremely dysphoric and well-remembered dreams that involve threats to survival, security, or physical integrity. Upon awakening from dysphoric dreams subjects are quickly oriented and alert, and the dream experience or sleep disturbance due to the awakening causes distress or impairment of social, occupational or other important areas of functioning.

Nightmares can occur as a single complaint or in the context of other disorders (Högl and Iranzo, 2017), such as psychiatric disorders (Swart et al., 2013), RBD (Schenck et al., 1987, Valli et al., 2012, Valli et al., 2015) or narcolepsy (Pisko et al., 2014). Frequent nightmares are uncommon and occur in 1–5 % of preadolescent children, and recurrent nightmares in childhood represent the best predictor of recurrent nightmares at an older age. Nightmares are reported as a problem by 2–8 % of the general population and are present in up to 80 % of patients with posttraumatic stress disorder.

### 4.2 Neurophysiology of nightmare disorder

An early neurophysiological study of nightmares was published in 1968. Broughton stated that nightmares occur in “confusional states of arousal” and not in “dreaming sleep” (Broughton, 1968). Later, the occurrence of nightmares during REM sleep, particularly during long REM periods occurring in the last part of the night or during REM periods with increased eye movements, was reported (Hartmann, 1984). This was in line with a previous report that nightmares arising from REM sleep showed an increase in the cardiorespiratory rate and in the number of eye movements (Fisher et al., 1970). Autonomic changes in nightmare sufferers included increases in respiratory and heart rate, and reduced heart rate variability (Fisher et al., 1970, Nielsen et al., 2010, Nielsen and Zadra, 2000).

One later study about recurrent nightmares performed EEG and auditory evoked potentials and included 10 nightmare sufferers and 10 controls. EEG was normal in all people with nightmares, and no differences in auditory evoked potentials between patients and controls were reported. N100, P160 and N200 were higher in amplitude in people with than without nightmares, but this difference was not statistically significant. Nightmare sufferers had less sleep, more awakenings and reduced slow wave sleep compared to published sleep norms (Newell et al., 1992). In line with this, a recent polysomnography study in 17 individuals with frequent nightmares compared to 23 control subjects reported reduced sleep efficiency, increased wakefulness, reduced amount of slow wave sleep, increased nocturnal awakenings, especially from sleep stage 2, and longer duration of REM sleep in people with nightmares (Simor et al., 2012).

For what concerns the pathophysiology of nightmares, changes in REM sleep have been investigated. A study hypothesized heightened REM sleep propensity induced by REM sleep deprivation and investigated 14 nightmare sufferers. Participants showed instead lower REM propensity (Nielsen et al., 2010). Twelve patients with idiopathic nightmares without psychiatric comorbidities had more microarousals during REM sleep in VPSG recordings than sleepwalkers had (Perogamvros et al., 2015).

One EEG spectral power analysis in 19 subjects with nightmare disorder and 21 healthy subjects found increased relative high alpha (10–14.5 Hz) and fronto-central increases in high delta (3–4 Hz) power during REM sleep, and a trend of increased fronto-central low alpha (7.75–9 Hz) power in NREM sleep. The authors concluded that increased high alpha power in the REM periods of nightmare sufferers is a wake-like EEG feature during REM sleep, which might contribute to the pathophysiology of nightmare disorder (Simor et al., 2013b). The same group further investigated the relationship between nightmare disorder and altered sleep microstructure in NREM sleep. An altered sleep microstructure (analyzed via CAP during NREM sleep) was reported in nightmare sufferers, with a reduced amount of CAP A1 phase and increased A2 and A3 phases. These findings are consistent with decreased slow wave sleep in nightmare sufferers and reflect dysfunctional sleep-wake regulation and the imbalance between sleep-promoting and arousing mechanisms during NREM sleep (Simor et al., 2013a), likely leading to sleep instability. Another polysomnography-based study in 19 nightmare-disordered and 21 control subjects showed that nightmare sufferers’ sleep is mainly disturbed (it shows e.g. increased alpha power, increased heart rate variability) during NREM and REM transitions, and stabilize after REM episodes (Simor et al., 2014).

Nightmares frequently occur in posttraumatic stress disorder, and probably present different characteristics in these patients. A polysomnography study investigating a group of combat-veterans with posttraumatic stress disorder found high sleep efficiencies and an increase in wake after sleep onset in patients with trauma-related nightmares compared to other types of nightmares (Woodward et al., 2000). A further study used Script-driven imagery to assess nightmare imagery-evoked physiological and emotional reactivity in trauma exposed individuals with chronic nightmares. Nightmare imagery increased heart rate (Rhudy et al., 2008). Another study including 73 women with posttraumatic stress disorder (PTSD) found that self-reported nightmare severity was associated with greater heart rate response (Tanev et al., 2017).

Respiratory rate and its variability were measured in 49 Vietnam combat-related PTSD patients (11 with comorbid panic disorder and 38 without), and 15 controls. Respiration during sleep did not differ between PTSD and controls, but respiration during REM and NREM sleep was more rapid in PTSD patients with nightmares and/or comorbid major depressive disorder than in PTSD patients without such complaints. Of note, PTSD patients without nightmares breathed more slowly than controls (Woodward et al., 2003).

Additionally, there seems to be an association between nightmares and sleep apnea syndrome. Apnea-hypopnea-index (AHI) was polysomnographically measured in 20 veterans with PTSD and compared to 24 veterans without PTSD and to 17 healthy controls. An AHI > 10 / h occurred in 29 % PTSD, 21 % trauma controls and 29 % healthy controls, but the mean Clinician Administered PTSD Scale (CAPS) scores were significantly higher in patients with sleep apnea compared to those without, indicating that comorbid sleep related breathing disorder may worsen PTSD symptoms (van Liempt et al., 2011).

A possible link between obstructive sleep apnea (OSA) and nightmares seems to be present also in people without PTSD; as supported by a study in 99 patients with comorbid OSA and nightmares. OSA patients with nightmares had significantly higher AHI during REM sleep compared to OSA patients without nightmares. Nightmares disappeared in 91 % of patients treated with continuous positive airway pressure (CPAP)- therapy compared to 36 % who refused CPAP (BaHammam et al., 2013). However, another study found no association between OSA and nightmares (Schredl et al., 2006), thus further studies are needed to clarify this association.

## 5 Conclusions and future directions

Neurophysiology of RBD has been extensively studied. Common findings include EEG slowing, higher amount of delta power in EEG spectral power analysis, impaired functional connectivity, morphological differences in sleep spindles, slow oscillations and k-complexes, autonomic dysfunction, and abnormalities in evoked potentials and transcranial magnetic stimulation. In isolated RBD, neurophysiology data have been described as phenoconversion biomarker to clinically manifest alpha-synucleinopaty, and will likely gain more relevance in this context in the next future.

In contrast, neurophysiological data about sleep paralysis and nightmares are scarce and were mainly collected in studies with a small sample size. During sleep paralysis, EEG and VPSG studies reported the occurrence of a REM-wake dissociated stage, i.e. an intermediate state between REM and wakefulness, REM sleep or EEG activity with alpha frequencies. People with nightmare disorder showed less sleep, more awakenings, reduced slow wave sleep, increased respiratory and heart rate, and high alpha power in the REM periods, possibly indicating wake-like EEG features during REM sleep. Larger case-control EEG and VPSG studies investigating sleep paralysis and nightmare disorder are needed to better characterize the pathophysiology of both phenomena.

## Conflict of interest

None of the authors have potential conflicts of interest to be disclosed.

## Funding sources

This research did not receive any specific grant from funding agencies in the public, commercial, or not-for-profit sectors.

## CRediT authorship contribution statement

**Melanie Bergmann:** Writing – original draft, Writing – review & editing. **Birgit Högl:** Supervision, Conceptualization, Methodology, Writing – review & editing. **Ambra Stefani:** Supervision, Conceptualization, Methodology, Writing – review & editing.
